# Myocardial scarring on cardiovascular magnetic resonance in asymptomatic or minimally symptomatic patients with “pure” apical hypertrophic cardiomyopathy

**DOI:** 10.1186/1532-429X-14-52

**Published:** 2012-07-28

**Authors:** Kyung-Hee Kim, Hyung-Kwan Kim, In-Chang Hwang, Seung-Pyo Lee, Eun-Ah Park, Whal Lee, Yong-Jin Kim, Jae-Hyung Park, Dae-Won Sohn

**Affiliations:** 1 Division of Cardiology, Department of Internal Medicine, Cardiovascular Center, Seoul National University College of Medicine, Seoul National University Hospital, 28 Yongon-dong, Chongno-gu, Seoul, 110-744, South Korea; 2 Department of Radiology, Seoul National University College of Medicine, Seoul National University Hospital, 28 Yongon-dong, Chongno-gu, Seoul, 110-744, South Korea

**Keywords:** Apical hypertrophic cardiomyopathy, Cardiovascular magnetic resonance, Late gadolinium enhancement

## Abstract

**Background:**

Late gadolinium enhancement (LGE) cardiovascular magnetic resonance (CMR) enables state-of-the-art in vivo evaluations of myocardial fibrosis. Although LGE patterns have been well described in asymmetrical septal hypertrophy, conflicting results have been reported regarding the characteristics of LGE in apical hypertrophic cardiomyopathy (ApHCM). This study was undertaken to determine 1) the frequency and distribution of LGE and 2) its prognostic implication in ApHCM.

**Methods:**

Forty patients with asymptomatic or minimally symptomatic pure ApHCM (age, 60.2 ± 10.4 years, 31 men) were prospectively enrolled. LGE images were acquired using the inversion recovery segmented spoiled-gradient echo and phase-sensitive inversion recovery sequence, and analyzed using a 17-segment model. Summing the planimetered LGE areas in all short axis slices yielded the total volume of late enhancement, which was subsequently presented as a proportion of total LV myocardium (% LGE).

**Results:**

Mean maximal apical wall thickness was 17.9±2.3mm, and mean left ventricular (LV) ejection fraction was 67.7 ± 8.0%. All but one patient presented with electrocardiographic negative T wave inversion in anterolateral leads, with a mean maximum negative T wave of 7.2 ± 4.7mm. Nine patients (22.5%) had giant negative T waves, defined as the amplitude of ≥10mm, in electrocardiogram. LGE was detected in 130 segments of 30 patients (75.0%), occupying 4.9 ± 5.5% of LV myocardium. LGE was mainly detected at the junction between left and right ventricles in 12 (30%) and at the apex in 28 (70%), although LGE-positive areas were widely distributed, and not limited to the apex. Focal LGE at the non-hypertrophic LV segments was found in some ApHCM patients, even without LGE of hypertrophied apical segments. Over the 2-year follow-up, there was no one achieving the study end-point, defined as all-cause death, sudden cardiac death and hospitalization for heart failure.

**Conclusions:**

LGE was frequently observed not only in the thickened apex of the heart but also in other LV segments, irrespective of the presence or absence of hypertrophy. The simple presence of LGE on CMR was not representative of adverse prognosis in this population.

## Background

Apical hypertrophic cardiomyopathy (ApHCM) is a unique phenotype of hypertrophic cardiomyopathy (HCM) with a pathologic hypertrophy of the left ventricular (LV) apex and an “ace-of-spade” configuration of the LV cavity at end-diastole by echocardiography or cardiovascular magnetic resonance (CMR). Despite a relatively favorable prognosis for ApHCM, severe clinical manifestations including arrhythmias and life-threatening apical aneurysm have been occasionally reported [1]. Although transthoracic echocardiography has been traditionally used for diagnosing HCM, the diagnosis of ApHCM by echocardiography is sometimes challenging. In this special setting, CMR can provide additional, more accurate information on LV morphology [2]. In addition, the recent introduction of late gadolinium enhancement (LGE) CMR allows the accurate detection of myocardial fibrosis in an in vivo setting [3]. According to several earlier literatures, LGE is frequently observed in asymmetrical septal HCM patients, and is also closely associated with severe hypertrophy and a high risk of sudden cardiac death [3-6]. However, LGE features of CMR in ApHCM have rarely been reported, and moreover results disagree regarding LGE patterns in ApHCM [4,7,8]. Therefore, this study was set out to determine the frequency and distribution of LGE on contrast-enhanced CMR in ApHCM. In addition, based on close follow-up conducted over 2 years, we sought to determine whether LGE on CMR has prognostic impact in this population.

## Method

### Study population

In this prospective study, we enrolled 40 asymptomatic or minimally symptomatic ApHCM patients (31 men; average age 60.2 ± 10.4 years) scheduled for CMR in our institution. Diagnosis of ApHCM was established based on the demonstration of asymmetrical LV hypertrophy confined to the LV apex, an apical wall thickness ≥ 15mm, and a maximum apical thickness to maximum basal thickness ratio of ≥ 1.3 by CMR. Great care was taken to enroll ApHCM patients with involvement of the apex only [9,10]. All patients were evaluated with invasive or computed tomographic coronary angiography for the presence of coronary artery disease, and patients with significant stenosis, defined as luminal stenosis of > 50%, were systematically excluded. Patients who were not in sinus rhythm were excluded, as well. The study protocol was approved by the institutional review board of our hospital and informed consent was obtained from all participants before enrollment.

### Electrocardiography

A “giant” negative T wave in electrocardiogram (ECG) was defined as a voltage of negative T wave of ≥ 1 mV (≥10 mm) in any anterior lead. Maximum T wave inversion from leads V3, V4, V5, and V6 and summed T wave amplitudes in these leads were compared.

### Echocardiography

All echocardiographic examinations were performed on the same day as CMR using a commercially available machine (Vivid 7, GE, Horten, Norway) with subjects in the left lateral decubitus position. A routine standard echocardiographic examination, which included measurements of LV systolic and diastolic functions, was performed. In each case, maximal apical wall thickness at end-diastole was determined using the standard apical four-, two-, and three-chamber views.

### CMR protocol

CMR was performed using a 1.5T scanner (Sonata Magnetom, Siemens, Erlangen, Germany). Steady-state free precession cine images were used to quantify LV function (slice thickness of 6 mm), and LV short-axis images were acquired from apex to base to cover entire LV volumes. Repeated breath-holds were required in order to create adequate images. The temporal resolution was 25–30 frames per RR interval. LV mass was measured with commercially available software (QMASS MR; Medis, Leiden, Netherlands) by a single experienced observer unaware of echocardiographic data. LGE images were acquired 10 minutes after the intravenous administration of gadopentetatedimeglumine (0.15 mmol/kg intravenous injection, Magnevist; Schering, Berlin, Germany), and followed by a flush of 20mL of saline at the same rate. LGE images from long-axial, short-axial, and 4-chamber views were acquired using the inversion recovery segmented spoiled-gradient echo and phase-sensitive inversion recovery methods. LGE images were evaluated using a 17-segment model, as suggested by the American Heart Association criteria [11]. LV was evaluated in the short-axis images of basal, mid, and apical segments. Basal and mid cavities were divided into 6 equal segments: anterior, anteroseptal, inferoseptal, inferior, inferolateral, and anterolateral. Apical segments were divided into 4 segments: anterior, septal, inferior, and lateral. Apical cap constituted the 17^th^ segment of LV myocardium.

### Data analysis

**Figure 1:**
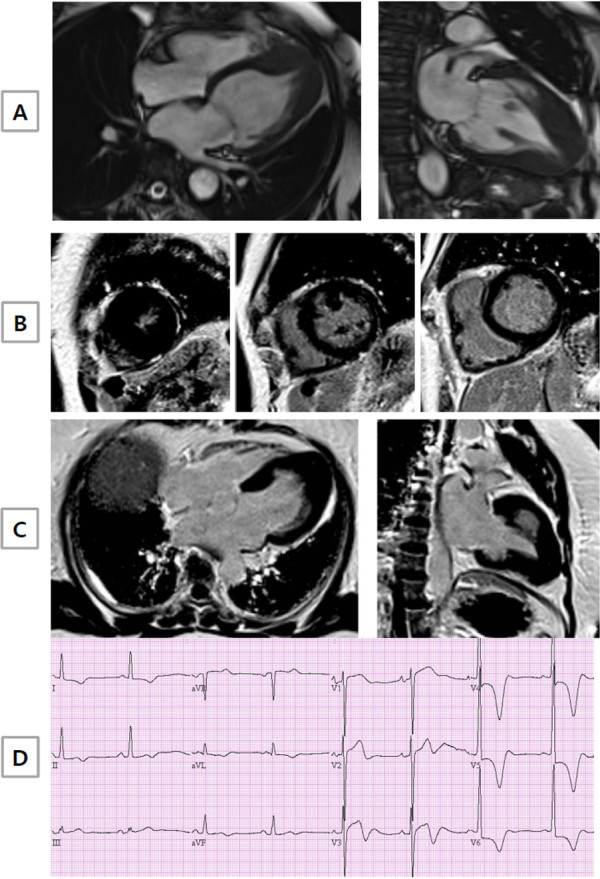
**A representative example of ApHCM without late gadolinium enhancement (LGE) by CMR.** (**A**) Long axis CMR cine images clearly demonstrated the presence of apical hypertrophy. However, short (**B**) and long axis (**C**) CMR images showed no LGE. (**D**) Giant negative T wave inversion in anterior leads of electrocardiogram was evident.

**Figure 2:**
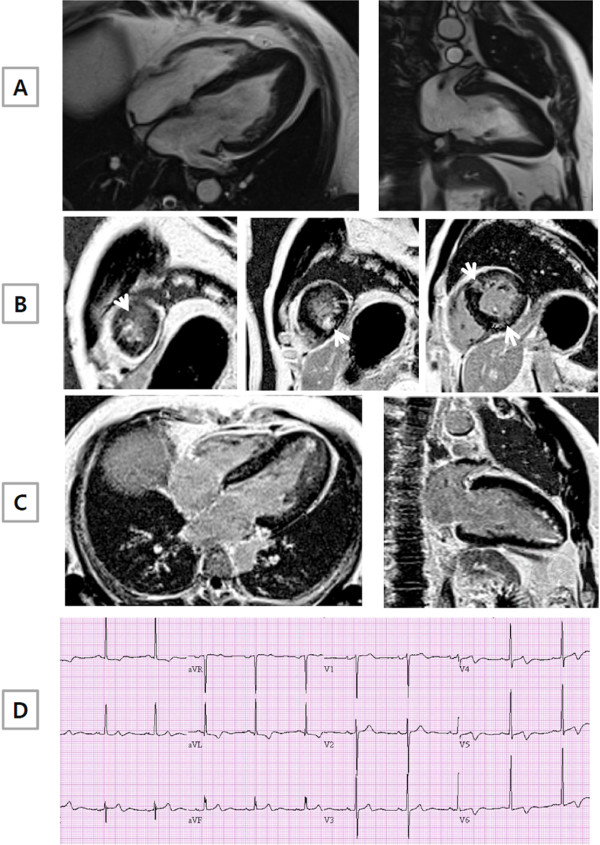
**A representative example of ApHCM with late gadolinium enhancement (LGE) by CMR.** (**A**) Long axis CMR cine images clearly demonstrated the presence of apical hypertrophy, as in Figure 1. In contrast, short (**B**) and long axis (**C**) CMR images showed diffuse LGE at the basal and mid interventricular septum or RV insertion site (non-hypertrophic segment) and at the apex (hypertrophic segment), as indicated by the arrow. (**D**) Negative T wave inversion in anterior leads of electrocardiogram was present.

Imaging data were analyzed with a commercially available postprocessing workstation (cmr42, Circle Cardiovascular Imaging Inc., Calgary, Canada). Endocardial and epicardial contours were traced manually on short-axis cine MR images of the LV at end diastole and end systole to assess end-diastolic and end-systolic volumes, LV ejection fraction, and LV mass. In order to assess the presence or absence of LGE and its extent, all short-axis slices from base to apex were inspected visually to identify areas of normal (completely nulled) myocardium. LGE was considered present when the signal intensity of the index myocardial segment was greater than 6SD, as compared with the remote normal myocardial signal [12,13]. Summing the planimetered areas of LGE in all short-axis slices yielded total volume of delayed enhancement, which was subsequently expressed as a proportion of total LV myocardium (% LGE). Contrast-enhanced images were analyzed by two experienced observers unaware of other CMR, echocardiographic, and clinical data (K.H.K., I.C.H.). Any discrepancy in analysis between the 2 readers was resolved by a senior observer (W.L.). Additionally, LGE in each of the 17 segments was graded using a 2-point scale (segmental fibrosis score; 0 = absence of LGE, 1 = presence of LGE), if CMR LGE was present using the 6SD method. Figures 1 and 2 are representative CMR examples of patients without and with diffuse LGE, respectively.

### Follow-up

In order to determine the impact of CMR-detected LGE on prognosis, ApHCM patients were clinically followed every 3 or 6 months for at least 2 years at a specialized HCM clinic by one cardiologist (K.H.K.). No patient was lost to follow-up. The study endpoint was a composite of all-cause mortality, sudden cardiac death, and hospitalization for heart failure.

### Statistical analysis

Data are presented as mean ± SD for continuous variables and as numbers (%) for categorical variables, as appropriate. After confirming the normality of the distributions of continuous variables using the Shapiro-Wilk test, the unpaired Student t-test was used to analyze normally distributed variables, and the Mann–Whitney *U* test to analyze non-normally distributed variables. Spearman correlation analysis was used to explore the possibilities of correlations between LV maximal wall thickness, ECG-determined T wave amplitudes. All statistical analyses were performed using statistical software package for windows (SPSS 17.0, SPSS Inc.) and a p value of < 0.05 was considered statistically significant.

## Results

### Clinical characteristics

Baseline clinical and demographic characteristics of the 40 ApHCM patients are illustrated in Table 1. Ages ranged from 28 to 80 years (median, 5 9years). There were 31 men and 9 women. Of the 40 patients, 25 (63.5%) were completely asymptomatic and 15 had only mild symptoms (New York Heart Association class I or II) at the time of study enrollment. The primary reason for initial echocardiography in the 25 asymptomatic patients was a general medical check-up or family screening. Three patients (7.5%) had a history of unexplained syncope. Although 17 patients (42.5%) had a history of treated hypertension, blood pressure had been well controlled for > 2 years in all. One patient (2.5%) had a family history of HCM and 3 patients (7.5%) had a family history of sudden death. All but one patient presented T wave inversion in anterolateral leads, with a mean maximum negative T wave amplitude of 7.8 ± 4.2mm. Of the 39 patients with T wave inversion on ECG, 12 (30%) presented giant negative T waves.

**Table 1:** Baseline clinical and electrocardiographic characteristics of the study population

	**N = 40**
Men/Women	31/9
Age	60.2 ± 10.4
Body surface area (g/m^2^)	1.74 ± 0.17
Family history of HCM	1(2.5%)
Family history of SCD	3 (7.5%)
History of syncope	3 (7.5%)
NYHA functional class I/II	25 (63.5%)/15 (37.5%)
Hypertension	17 (42.5%)
Diabetes mellitus	4 (10%)
Smoker	3 (7.5%)
Medications	
Calcium-channel blockers	13 (32.5%)
Beta blockers	9 (22.5%)
ACEi/ARB	10 (25%)
Aspirin	10 (25%)
Diuretics	6 (15%)
SBP/DBP	125.9 ± 13.7/75.9 ± 8.7
Electrocardiogram	
T wave inversion	39 (97.5%)
Giant T waves ≥10mm	12 (30%)

HCM denotes hypertrophic cardiomyopathy; SCD, sudden cardiac death; NYHA, New York Heart Association; ACEi/ARB, angiotension converting esterase inhibitor/angiotensin receptor blocker; S(D)BP, systolic (diastolic) blood pressure; ECG, electrocardiogram.

### Echocardiography

Echocardiographic variables are presented in Table 2. In 11 patients referred to our hospital, 2D echocardiogram performed at a local clinic reported mildly increased apical wall thickness (≥ 12mm), but it did not reach 15mm in most thickened apical segment even in the presence of negative T wave inversion. Based on echocardiography performed in our hospital, there was no patient who had an LV outflow tract obstruction, either at rest or after provocative valsalva maneuvers. Left atrial dimension was slightly increased (44.2 ± 4.8mm), suggestive of chronic LV diastolic dysfunction. Mean maximal apical wall thickness was 16.7 ± 2.4 mm. There was no significant correlation between maximal apical wall thickness and the amplitude of T wave inversion (r = 0.15, p = 0.34) or the sum of T wave amplitudes in precordial leads (r = 0.15, p = 0.36).

**Table 2:** Echocardiographic variables

LV end-diastolic dimension (mm)	48.3 ± 7.2
LV end-systolic dimension (mm)	27.3 ± 4.8
LV ejection fraction (%)	67.7 ± 7.2
IVS thickness (mm)	10.3 ± 1.6
PW thickness (mm)	9.8 ± 1.3
Maximal apical wall thickness at end-diastole (mm)	16.7 ± 2.4
LA end-systolic dimension (mm)	44.2 ± 4.8
Peak E wave velocity (m/s)	0.58 ± 0.12
Peak A wave velocity (m/s)	0.54 ± 0.16
Early mitral annular velocity (E’, m/s)	0.11 ± 0.42
Mitral inflow/annular velocity ratio (E/E’)	12.96 ± 4.4

LV denotes left ventricle; IVS, interventricular septum; PW, posterior wall; and LA, left atrium.

### CMR

**Figure 3:**
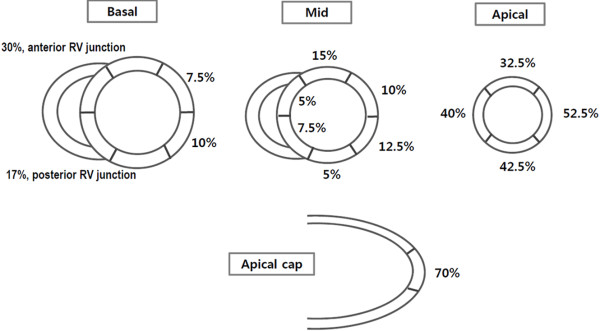
**Distribution of LGE on CMR in ApHCM patients.** In most patients, myocardial LGE was localized in the apicolateral and apical cap segments (52.5%, 70.0% respectively).

**Figure 4:**
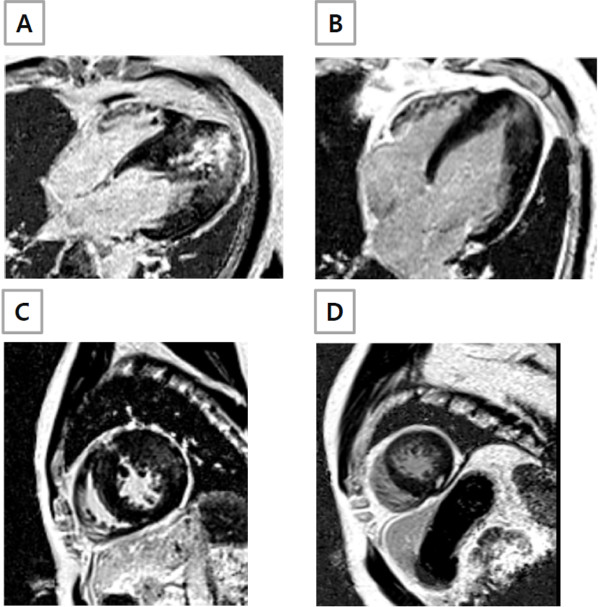
**LGE patterns observed in ApHCM patients.** (**A**) Subendocardial LGE with transmurality of ≥75%; (**B**) Fuzzy and confluent LGE; (**C**) Multifocal LGE at the RV insertion into the septum and mid anterior wall; (**D**) Discrete (focal) pattern at mid myocardium.

In total, 680 segments were analyzed for LV wall thickness and LGE on CMR. Mean LV mass index was 102.2 ± 28.9gm/m^2^ and LV ejection fraction was 67.7 ± 8.0% (Table 3). Maximal apical wall thickness ranged from 15mm to 26mm (17.9±2.3mm), which was significantly greater than that obtained by echocardiography (p < 0.01). Contrast images demonstrated LGE representing myocardial fibrosis in 30 (75.0%) of the 40 patients and in 130 (19%) of the 680 segments. Total volume of LGE was estimated to be 9.7 ± 10mL, corresponding to 4.9 ± 5.5% (rage 0.5% to 26%) of total LV myocardium. The most frequently involved myocardial segments were the apical cap (28 patients (70%)), followed by the apicolateral segment (20 patients (52.5%)). Of note, LGE was observed not only in the apex of the heart but also in the other segments of the LV, especially in mid anterior and anterolateral segments of the LV and at anterior and posterior attachment points of the right ventricle to the interventricular septum. In addition, LGE was detected in hypertrophied as well as non-hypertrophied areas of the heart. LGE distribution is shown schematically in Figure 3. Areas of LGE were transmural (≥75% of any segmental wall thickness) in only 2 (7%) of the 30 patients, exclusively limited to the apex and were nontrasmural in the other 28 (93%) patients, in focal, multifocal or confluent patterns. Interestingly, LGE observed at apical segments was in a “subendocardial” pattern (47 (48%) of 97 apical segments showing LGE), similar to LGE found in ischemic cardiomyopathy. Representative CMR images of LGE patterns are illustrated in Figure 4.

**Table 3:** CMR findings

LV end-diastolic volume (mL)	130.5 ± 26.3
LV end-diastolic volume index (mL/m^2^)	76.0 ± 13.9
LV end-systolic volume (mL)	43.9 ± 18.1
LV end-systolic volume index (mL/m^2^)	25.2 ± 9.1
LV ejection fraction (%)	67.7 ± 8.0
LV mass (gm)	178.0 ± 61.1
LV mass index (gm/m^2^)	102.2 ± 28.9
Maximal apical wall thickness at end-diastole (mm)	17.9 ± 2.3

LV denotes left ventricle.

Although no significant correlation between the depth of T wave inversion on ECG and maximal apical thickness on echocardiography could be demonstrated, maximal apical wall thickness determined by CMR displayed a modest but significant correlation with the amplitude of T wave inversion on ECG (r = 0.38, p = 0.016). An apical aneurysm was detected in 5 patients (12.5%), and 3 of these (60%) were not detected by routine echocardiography.

### Follow-up

Over the 2-year follow-up, there was no one who achieved the study end-point, defined a priori, as all-cause death, sudden cardiac death and hospitalization for heart failure. Arrhythmic events that were assessed in 23 patients who underwent 24-hour Holter monitoring both at baseline and follow-up were also unremarkable and thus could not be predicted by CMR LGE patterns or its extent, either.

## Discussion

Japanese investigators first reported ApHCM as a morphologic variant of HCM characterized by a striking electrocardiographic pattern of deep T wave inversion in precordial leads and a distinctive angiographic “ace-of-spades” like appearance of the LV at end-diastole [14-16]. Subsequently, echocardiographically detected ApHCM has been repeatedly reported at several centers outside of Japan, although the characteristic electrocardiographic patterns are reported inconsistently in Western patients [1,17]. In the present study, in accordance with previous observations [1,18], all but 1 patient (97.5%) presented with electrocardiographic alterations of ventricular repolarization, in the form of T wave inversion. The prevalence of a giant negative T wave in our population is also similar to that reported in a Canadian study [1].

Two-dimensional echocardiography is now generally regarded as the “*clinical standard*” noninvasive diagnostic test for HCM [19,20]. However, conventional echocardiography has its technical limitations. As shown in a previous study, it tends to underestimate the magnitude of hypertrophy in the anterolateral free wall, which is frequently spatially away from the center of the echocardiographic sector in parasternal short axis view, resulting in poor lateral resolution, and inconclusive delineation of the epicardial border [21]. Another pitfall of conventional echocardiography is that procurement of apical wall images is not easy due to the close proximity of the echocardiographic probe and the apex, which may result in missing the diagnosis of ApHCM [22,23]. In a previous study, CMR was compared with echocardiography in terms of its ability to detecting hypertrophied segments, and it was concluded that CMR is superior to echocardiography, especially with respect to the detection of apical segmental hypertrophy [24]. In line with findings of the previous study [24], we found that echocardiography significantly underestimated apical wall thickness, as compared with CMR (16.7 ± 2.4 mm vs. 17.9 ± 2.3 mm, p < 0.01). Furthermore, echocardiograms performed at a local clinic did not demonstrate sufficient LV apical wall thickness for objective evidence of diagnosing ApHCM in 11 out of 40 patients, that is, in these patients apical wall thickness were < 15mm even in the presence of electrocardiographic T wave inversion, highlighting the advantage of CMR over conventional echocardiography in the accurate delineation of the epicardial border. The advantage of CMR with respect to the visualization of hypertrophied apical LV segments, sometimes not readily identifiable by conventional echocardiography, has been previously demonstrated in ApHCM [2]. The significant correlation found in the present study between the amplitude of T wave inversion on ECG and maximal apical wall thickness measured by CMR, but not by echocardiography, confirms the advantage of CMR over echocardiography for the assessments of apical pathology.

In the present study, we found that myocardial fibrosis is not an uncommon finding in even asymptomatic or mildly symptomatic ‘pure’ ApHCM patients. Furthermore, we observed that apical segments, including apical cap, were the most frequently involved LGE sites in ApHCM patients. However, we found that LGE was not limited to hypertrophic apical segments, and that it was also present in basal segments, at the anterior and posterior junctions between the septum and RV free wall, and additionally in non-hypertrophied segments of ApHCM (Figure 3). More importantly, LGE observed at apex (including apical cap) (47 (48%) of 97 apical segments showing LGE) was in a “subendocardial” pattern, quite unlike the patchy or focal mid-wall LGE pattern frequently observed in asymmetrical septal HCM patients [4,5,8]. Given that “*ischemic-type*” LGE is usually characterized by subendocardial involvement with or without extension into subepicardium [25], the subendocardial LGE pattern at apex in our population strongly suggests previous subendocardial ischemia. Since we exclusively recruited ApHCM patients without significant epicardial coronary artery disease, this “*ischemic-type*” LGE should be attributable to mechanisms other than epicardial coronary stenosis. A hypertrophied apical wall with relatively deficient coronary blood flow may be a plausible explanation for this “*ischemic-type*” LGE. However, an increment in intra-apical cavitary pressure that might subsequently lead to suboptimal perfusion to the subendocardial area could explain “*ischemic-type*” LGE at the apex, as well [26].

Although few studies have addressed LGE in ApHCM, spatial distributions of LGE in ApHCM disagree. In one earlier report, LGE was described only at the apex without any involvement of the base or mid ventricular levels [7], whereas in another report LGE was observed widely in ApHCM patients. More recently, Amano et al. showed the presence of LGE at apex as well as mid-ventricular segments in ApHCM patients [27]. However, they only enrolled symptomatic patients, and thus we still have no data regarding whether LGE is widely distributed even in asymptomatic or minimally symptomatic ApHCM patients. In fact, our study is the largest to investigate the extent and distribution of LGE in an asymptomatic or minimally symptomatic subset of ApHCM, and based on our finding, LGE appears to be distributed across all myocardial segments even in the asymptomatic or minimally symptomatic ApHCM population.

Although ApHCM patients enrolled in the current study were carefully followed for at least 2 years, no one achieved the study end-point (all-cause death, sudden cardiac death or hospitalization for heart failure). No significant arrhythmic events were found, either. Due to the relatively short follow-up period, however, definite conclusions cannot be drawn.

Some limitations of this study warrant consideration. First, the presence of LGE on contrast-enhanced CMR has been well validated in animal models and in humans with ischemic heart disease [27,28]. Although LGE on CMR in HCM patients has been shown to represent increased myocardial collagen in one HCM patient with heart failure, extensive evaluation regarding validation of LGE on CMR has not been systematically performed in ApHCM patients, and thus, the view that LGE exclusively reflects myocardial scarring is, more or less, speculative. Second, the lack of long-term longitudinal follow-up data prevented definite conclusion of prognostic impact of LGE on CMR in this subset of ApHCM patients. Same caution should be exercised with regard to the prediction of arrhythmic events in this population.

## Conclusions

LGE on CMR is not an uncommon finding in ApHCM patients, and is widely distributed across almost all LV myocardial segments, including non-hypertrophied segments. Given no morbidity or mortality during 2 years of follow-up, it would appear that the simple presence of LGE on CMR is unlikely to be representative of adverse prognosis in this asymptomatic or minimally symptomatic ApHCM population, esp. in the short term.

## Abbreviations

ApHCM, Apical hypertrophic cardiomyopathy; HCM, Hypertrophic cardiomyopathy; CMR, Cardiovascular magnetic resonance; LGE, Late gadolinium enhancement; LV, Left ventricle.

## Competing interest

The authors declare that they have no competing interests.

## Authors’ contributions

K-HK, H-KK, E-AP, WL, and J-HP contributed to the study design, and performed the analysis. H-KK, S-PL, Y-JK, and D-WS recruited patients and interpreted data. I-CH significantly contributed to LGE assessment of CMR and also interpretation of the data during revision period. K-HK and H-KK wrote the manuscript. All authors have read and approved the final manuscript.
